# Advancement of pharmacokinetic models of iohexol in patients aged 70 years or older with impaired kidney function

**DOI:** 10.1038/s41598-021-01892-1

**Published:** 2021-11-22

**Authors:** Max Taubert, Elke Schaeffner, Peter Martus, Markus van der Giet, Uwe Fuhr, Amina Lösment, Natalie Ebert

**Affiliations:** 1 Clinical Pharmacology Unit, Department I of Pharmacology, Faculty of Medicine and University Hospital Cologne, Center for Pharmacology, University Hospital Cologne (AöR), Gleueler Straße 24, 50931 Cologne, Germany; 2 Institute of Public Health, Charité – Universitätsmedizin Berlin, Berlin, Germany; 3 Institute for Clinical Epidemiology and Applied Biostatistics, University Hospital Tübingen, Tübingen, Germany; 4 Department of Nephrology, Charité – Universitätsmedizin Berlin, Berlin, Germany; 5 Department of Nephrology, Vivantes Klinikum Im Friedrichshain, Berlin, Germany

**Keywords:** Nephrology, Kidney, Kidney diseases

## Abstract

Plasma clearance of iohexol is a pivotal metric to quantify glomerular filtration rate (GFR), but the optimal timing and frequency of plasma sampling remain to be assessed. In this study, we evaluated the impact of a Bayesian estimation procedure on iohexol clearance estimates, and we identified an optimal sampling strategy based on data in individuals aged 70+. Assuming a varying number of random effects, we re-estimated previously developed population pharmacokinetic two- and three-compartment models in a model development group comprising 546 patients with iohexol concentration data up to 300 min post injection. Model performance and optimal sampling times were assessed in an evaluation group comprising 104 patients with reduced GFR and concentration data up to 1440 min post injection. Two- and three-compartment models with random effects for all parameters overestimated clearance values (bias 5.07 and 4.40 mL/min, respectively) and underpredicted 24-h concentrations (bias − 14.5 and − 12.0 µg/ml, respectively). Clearance estimates improved distinctly when limiting random effects of the three-compartment model to clearance and central volume of distribution. Two blood samples, one early and one 300 min post injection, were sufficient to estimate iohexol clearance. A simplified three-compartment model is optimal to estimate iohexol clearance in elderly patients with reduced GFR.

## Introduction

Plasma clearance of iohexol has become a pivotal metric to quantify glomerular filtration rate (GFR) in clinical practice and in research since iohexol is almost exclusively eliminated by the kidneys and has proven to be safe^1^. However, a standardized and universally accepted protocol for iohexol clearance measurement is still missing, which is an essential prerequisite for routine use^2^. The lack of protocol standardization is partly since there is no agreement on the optimal blood sampling times and the choice of a pharmacokinetic model and estimation method. Different approaches have been suggested, ranging from curve-stripping^3^ to compartmental models^4,5^. In a recent investigation, we have shown that a three-compartment non-linear mixed-effects model significantly improved the description of iohexol pharmacokinetics compared to a two-compartment approach by accounting for an early and rapid distributional component of iohexol pharmacokinetics^6^.

Despite obvious advantages at the population level, it remains unclear whether the use of a three-compartment model results in a clinically relevant improvement of individual iohexol plasma clearance estimates. A potential disadvantage of three-compartment non-linear mixed-effects models is the need for an empirical Bayesian estimation procedure when estimating individual pharmacokinetic (PK) parameters. When using an empirical Bayesian estimation procedure, a population PK model serves as a prior distribution to inform the estimation of individual parameters, which may result in bias. For example, clearance estimates can be inadequately shifted towards the typical clearance in the population if a patient’s characteristics differ significantly from the source population or if patient data are incomplete.

Furthermore, different protocols with regard to the number and timing of iohexol plasma samples have been suggested, ranging from a single sample^7^ to extensive sampling^8^, including samples taken up to 1440 min after iohexol administration in patients with low GFR^9^.

The aim of the current investigation is to evaluate the impact of the Bayesian estimation procedure on iohexol clearance estimation, and to identify the optimal sampling strategy based on data in individuals aged 70+.

## Methods

The present analysis is based on data from the previously conducted Berlin Initiative Study (BIS)^10,11^, which was approved by the Ethics Committee of Charité Universitätsmedizin Berlin (proposal number EA2/009/08) and carried out in full agreement with all relevant regulations. All participants gave written informed consent.

Inclusion criteria for iohexol plasma clearance measurement, a sub-study of the BIS, were age 70 and above, thyroid-stimulating hormone level > 0.3 mIU/L, and no known iodine allergy. In the original sample used to develop the BIS equations, 570 patients were included^10^. Data from seven patients were excluded from this analysis due to implausible concentration–time profiles. Out of the remaining 563 patients, samples obtained 1440 min post injection were available for 17 patients resulting in a subset of 546 patients with data obtained up to 300 min post injection and without implausible concentration–time profiles. This subset with data from 546 patients served as the model development group, while data from the 17 patients with samples obtained up to 1440 min post injection were included in the evaluation group. The evaluation group was complemented by data from 87 patients with considerably reduced kidney function who underwent iohexol plasma clearance measurements including plasma samples obtained 1440 min post injection referred to us from specialist outpatient care. Consequently, the final evaluation group includes 104 patients with samples obtained 1440 min post injection^11^. The selection process for the study population is presented in a flow chart (Supp. Fig. 1). Iohexol plasma concentrations from 104 patients after intravenous bolus administration of 3235 mg iohexol were quantified at 30, 60, 90, 120, 150, 180, 240, 300 and 1440 min using high-performance liquid chromatography as described previously^10,11^. The statistical analysis was carried out using NONMEM 7.4.3^12^ and R software^13^ with the tidyverse^14^, vpc^15^, and nonmemica^16^ packages.

This investigation includes three parts. In the first part, we implement population pharmacokinetic (PK) two- and three-compartment models that were previously developed based on iohexol concentrations up to 300 min post injection^6^ and re-estimate fixed effects parameters based on data from the model development group including 546 patients. Based on the resulting models, we obtained empirical Bayes estimates (EBEs; maximum a posteriori estimates) of elimination clearance from the 104 patients of the evaluation group not including concentrations observed 1440 min post injection. In a second step, concentrations 1440 min post injection were predicted based on EBEs and compared to observed concentrations in terms of root mean squared error (RMSE, Eq. 1). Furthermore, the impact of including versus omitting observations 1440 min post injection on clearance EBEs was quantified in terms of clearance RMSE (Eq. 2). Therefore, concentrations observed 1440 min post injection were used as the reference values for assessing predicted concentrations, and the calculated iohexol clearances of patients based on all available iohexol plasma concentrations up to 1440 min post injection were used as the reference values for assessing clearance values determined based on a restricted number of iohexol samples up to 300 min post injection. Additionally, Lin’s concordance correlation coefficient (CCC)^17^, the relative total deviation index (TDI) for a range of coverage probabilities (CP)^18^, and the percentage of patients with relative differences not exceeding 10% (P10) or 30% (P30) of the reference clearance values were evaluated^19^. A TDI of ≤ 10% for a CP of 90%, based on observed relative differences between iohexol clearance values given a restricted number of samples up to 300 min post injection and iohexol clearance reference values based on all available samples, was considered optimal. A CCC value of > 0.99, 0.95 to 0.99, 0.90 to 0.95 or < 0.9 was considered to reflect almost perfect, substantial, moderate or poor concordance^20,21^. An at least substantial concordance was considered optimal.

1 $${\text{RMSE}}_{{{\text{conc}}}} = \sqrt {\frac{1}{104}\mathop \sum \limits_{i = 1}^{104} \left( {Cp\left( {t = 1440 \min } \right) - \widehat{Cp}\left( {t = 1440 \min } \right)} \right)^{2} }$$

Equation (1) Root mean squared error ($RMS{E}_{conc}$) concerning observed ($Cp\left(t=1440 \, {\rm min}\right)$) versus predicted ($\widehat{Cp}\left(t=1440 \, {\rm min}\right)$) iohexol concentrations 1440 min post injection. Predicted concentrations were based on clearance EBEs obtained including iohexol concentration data up to 300 min post injection, i.e., by ignoring concentrations observed after 1440 min in the EBE estimation.

2 $${\text{RMSE}}_{CL} = \sqrt {\frac{1}{104}\mathop \sum \limits_{i = 1}^{104} \left( {CL_{1440 \min } - CL_{300 \min } } \right)^{2} }$$

Equation (2) Root mean squared error ($RMS{E}_{CL}$) concerning clearance EBEs obtained when including observations up to 1440 min post injection ($C{L}_{1440 \min}$) compared to clearance EBEs obtained based on data up to 300 min post injection ($C{L}_{300 \min}$).

In the second part, we restricted the random effects (RE) of the two- and three-compartment models to the elimination clearance (CL) and the central volume of distribution (V1). Fixed effects parameters were re-estimated based on data from the 546 patients in the model development group and the resulting models were compared to models with random effects for all PK parameters in terms of RMSE as described above. Furthermore, age, sex, body weight, serum creatinine (CR), and serum cystatin C (CC) concentrations were evaluated as clearance covariates and body weight as a covariate on the volume of distribution based on the model that provided the lowest RMSE. For this purpose, the following covariate equations (Eqs. 3 and 4) were applied, in which CL and V1 in the i^th^ subject are based on the typical value in the population (${\theta }_{1}, {\theta }_{7}$) and a combination of covariate effects with parameter estimates represented by ${\theta }_{2}$ to ${\theta }_{6}$ and ${\theta }_{8}$. In these equations, covariates were normalized to the respective median in the group of patients for computational reasons. CCC, TDI_90_, P10 and P30 were considered as additional measures analogous to part 1. Finally, we used the BIS2 equation^6,10^ to calculate the estimated GFR (eGFR) values based on creatinine and cystatin C data to identify relationships between eGFR and RMSE for different models.

3 $$CL_{i} = \theta_{1} \times \left( {\frac{CR}{{0.9}}} \right)^{{\theta_{2} }} \times \left( {\frac{CC}{{1.03}}} \right)^{{\theta_{3} }} \times \left( {\frac{Age}{{77}}} \right)^{{\theta_{4} }} \times \left( {\frac{{{\text{Weight}}}}{77}} \right)^{{\theta_{5} }} \times (1 + \theta_{6} ) \left( {{\text{if}}\,{\text{female}}} \right)$$

Equation (3) Individual clearance in the ith subject ($C{L}_{i}$) based on the covariates serum creatinine (CR), cystatin C (CC), age, body weight, and sex. ${\theta }_{1}$ is the typical (median/geometric mean) clearance in the population while ${\theta }_{2}$ to ${\theta }_{6}$ represent the coefficients associated with covariate effects.

4 $$V1_{i} = \theta_{7} \times \left( {\frac{{{\text{Weight}}}}{77}} \right)^{{\theta_{8} }}$$

Equation (4) Individual central volume of distribution in the ith subject ($V{1}_{i}$) based on body weight. ${\theta }_{7}$ is the typical (median/geometric mean) central volume of distribution while ${\theta }_{8}$ represents the coefficient associated with the effect of body weight.

Subsequently, a power prior approach^22^ was used to evaluate the impact of the Bayesian procedure on clearance EBEs for a selection of models. EBEs are typically obtained by finding the vector of random effects (${{\varvec{\eta}}}_{{\varvec{i}}}$) that minimizes the objective function value given in Eq. (5) with $\phi =1$. Equation 5 represents a combination of the likelihood given the *j*th observation in the *i*th subject (${y}_{ij}$), the corresponding model-based predicted value ($IPRE{D}_{ij}$) and the residual error variance of observations (${\sigma }_{ij}^{2}$), as well as the prior probability of ${{\varvec{\eta}}}_{{\varvec{i}}}$ given the covariance matrix of random effects (${\varvec{\Omega}}$). Random effects are assumed to follow a multivariate normal distribution with mean zero, and they are transformed into individual pharmacokinetic parameters ($P{K}_{i}^{p}$) via $P{K}_{i}^{p}=P{K}_{pop}^{p}\times \mathrm{exp}\left({\eta }_{i}^{p}\right)$. Here, $P{K}_{pop}^{p}$ is the geometric mean of the *p*th pharmacokinetic parameter in the population, and ${\eta }_{i}^{p}$ is the corresponding random effect in the *i*th subject. By adjusting the parameter $\phi$, the impact of the population model on the estimation procedure can be altered systematically. For example, the minimization of Eq. (3) with $\phi =0$ represents maximum likelihood while $\phi =1$ represents classical EBE estimation.

5 $$OBJ_{i} = \mathop \sum \limits_{j} \left( {\log \left( {\sigma_{ij}^{2} } \right) + \frac{{\left( {y_{ij} - IPRED_{ij} } \right)^{2} }}{{\sigma_{ij}^{2} }}} \right) + \phi \eta_{i} \Omega^{ - 1} \eta_{i}^{T}$$

Equation (5) Objective function value for the *i*th subject ($OB{J}_{i}$) based on the *j*th observation (${y}_{ij}$), the corresponding model-based predicted value ($IPRE{D}_{ij}$), the residual error (${\sigma }_{ij}^{2}$) and the prior probability of the vector of random effects ${{\varvec{\eta}}}_{{\varvec{i}}}$ given the covariance matrix of random effects (${\varvec{\Omega}}$).

In the third part, we identified optimal sampling times and the optimal number of samples by evaluating EBEs for all possible combinations of one to four plasma samples for each of the 104 patients in the evaluation group. The optimal sampling protocol was defined as the combination of sampling times resulting in the lowest overall RMSE of predicted concentrations 1440 min post injection and individual clearance estimates. For the latter, we used clearance estimates obtained when including all available iohexol samples up to 1440 min post injection as reference values. Furthermore, we carried out a stochastic simulation to evaluate optimal sampling times for each model separately by comparing simulated to estimated individual clearance values based on all possible combinations of one to four samples. For this purpose, iohexol concentrations were simulated 30, 60, 90, 120, 180, 240, and 300 min post injection in 6,500 subjects (ten times the number of the 546 plus 104 patients) by sampling random effects from a multivariate normal distribution with (co-)variances obtained from the respective population PK model. CCC, TDI_90_, P10 and P30 were utilized to further compare the performance of sampling schedules depending on a varying number of iohexol samples analogous to parts 1 and 2.

## Results

Patients evaluated in the present investigation had similar age and weight but lower eGFR levels compared to BIS participants included in our previous study^6^ (Table 1). The current patient population comprised a larger fraction of female subjects (67% vs. 34%) and showed (by definition) systematically higher serum creatinine and cystatin C concentrations, indicating a lower average kidney function.

**Table 1:** Characteristics (mean [standard deviation] or absolute frequency [%]) of the study population compared to the previously investigated study population with only 300 min of iohexol sampling time.

	Evaluation group with observations up to 1440 min post injection	Model development group with observations up to 300 min post injection
Number of subjects	104	546
Female, n (%)	34 (33%)	238 (44%)
Age (years)	79.2 (6.09)	78.3 (6.03)
Body weight (kg)	79.5 (13.9)	77.3 (13.9)
BSA (m^2^)	1.89 (0.185)	1.85 (0.187)
Serum creatinine (mg/dL)	1.91 (0.603)	0.962 (0.289)
Serum cystatin C (mg/L)	2.13 (0.745)	1.11 (0.309)
eGFR_BIS2_ (mL/min/1.73m^2^)	29.4 (7.86)	54.9 (12.3)

The population pharmacokinetic models were developed in a study population of n = 546 (model development group) and validated in a study population with iohexol sampling up to 1440 min (n = 104, evaluation group). eGFR = estimated glomerular filtration rate, BIS2 = eGFR equation from the Berlin Initiative Study based on serum creatinine and cystatin C. Body surface area (BSA) based on the Du Bois & Du Bois equation ($BSA= 0.20247*heigh{t}^{0.725}*weigh{t}^{0.425}$).

### Part I—evaluation of models with random effects for all PK parameters

**Figure 1:**
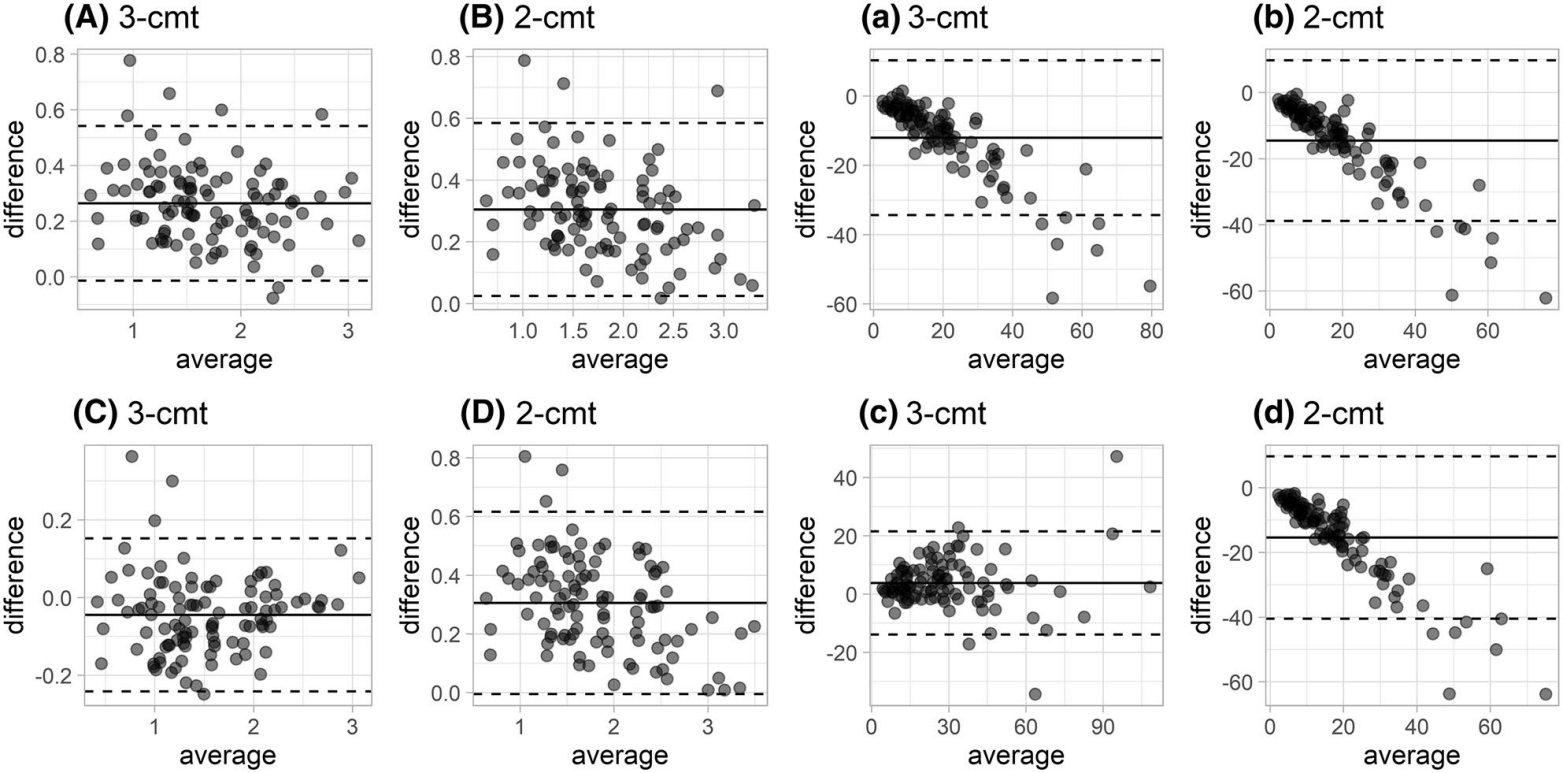
Bland–Altman plots comparing clearance estimates obtained without versus with iohexol concentrations observed 1440 min post injection (**A**, **B**, **C**, **D**) as well as predicted versus observed concentrations 1440 min post injection (**a**, **b**, **c**, **d**) based on empirical Bayes estimates given concentrations ≤ 5-h post injection. Random effects for all pharmacokinetic parameters of three (3-cmt; **A** and **a**) and two (2-cmt; **B** and **b**) compartment models and with random effects restricted to CL and V1 with three (**C** and **c**) and two (**D** and **d**) compartment models.

The models with random effects for all PK parameters provided overestimated clearance values and underpredicted concentrations 1440 min post injection irrespective of the use of two or three compartments. We found that bias and RMSE were similar in magnitude concerning clearance (bias of 5.07 and RMSE of 5.60 mL/min for the two-compartment; 4.40 and 4.99 mL/min, respectively, for the three-compartment model). CCC values for the two- and three-compartment models were 0.86 and 0.87, respectively, indicating a poor concordance between clearance estimates and reference values (Supp. Table 2). Accordingly, the TDI_90_ indicated that the iohexol clearance estimates deviated from the reference values by at least 40 and 45%, respectively, in 10% of the patients. Similarly, P10 and P30 covered only a limited fraction of patients (Supp. Table 2). Please refer to Supp. Fig. 3 for the empirical distribution of CP versus TDI values. The bias concerning clearance was more pronounced at lower clearance values for both models (Fig. 1). With respect to iohexol concentrations 1440 min post injection, we found a bias and RMSE of − 14.5 and 19.0 µg/ml for the two-compartment and − 12.0 and 16.5 µg/ml for the three-compartment model, respectively.

### Part II—evaluation of models with reduced random effects structures

The RMSE and the bias for iohexol clearance and for concentrations 1440 min post injection of the three-compartment model could be significantly reduced when restricting random effects to CL and V1 (RMSE of 1.82 mL/min and 9.72 µg/ml, respectively; bias of -0.742 mL/min and 3.78 µg/ml, respectively) (Fig. 1). In contrast, no relevant improvement was observed for the two-compartment model (RMSE of 5.73 mL/min and 20.0 µg/ml, respectively; bias of 5.10 mL/min and − 15.4 µg/ml, respectively). The inclusion of the covariates shown in Table 1 (gender, age, body weight, creatinine and cystatin C) provided a distinctly reduced objective function value based on data from the model development group with only 300 min iohexol sampling time (OFV reduced by 1320 points), and a slight improvement in terms of RMSE and bias when applying the model with covariates to data from the evaluation group. While the two-compartment model with limited random effects was not relevantly better compared to the full two-compartment model in terms of CCC and TDI_90_, a clear improvement was observed in case of the three-compartment models (Supp. Table 2). For the limited three-compartment model without covariates, the CCC increased to 0.98 and the TDI_90_ reduced to 15%. A further improvement was observed when including covariates, resulting in a CCC of 0.99 and a TDI_90_ of 12%. Thus, the concordance was considered substantial based on the CCC, while the pre-defined optimal goal in terms of TDI_90_ was not achieved. Relative deviations exceeding 10% were predominantly observed in patients with a model-estimated iohexol clearance < 20 mL/min, for whom the TDI_90_ was 19 and 18% for the model without and with covariates, respectively. In contrast, the TDI_90_ was 9.0 and 7.4% in patients with an estimated clearance of ≥ 20 mL/min, reaching the pre-defined TDI_90_ goal. Please refer to Supp. Fig. 3 for the empirical distribution of CP versus TDI values.

**Figure 2:**
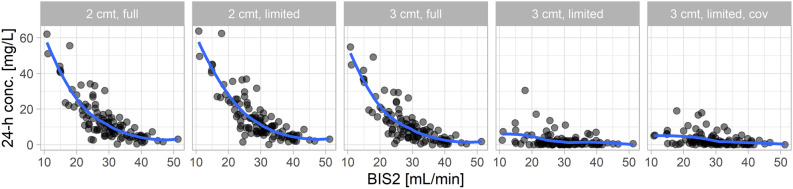
Absolute difference between predicted and observed 1440 min concentrations (dots) versus eGFR values as estimated with the creatinine and cystatin C-based BIS2 equation for the five evaluated models. Two- (2 cmt) and three-compartment (3 cmt) models with random effects for all PK parameters (full) or restricted to CL and V1 (limited). One model with covariates (cov). LOESS (locally estimated scatterplot smoothing) curve (blue line).

**Figure 3:**
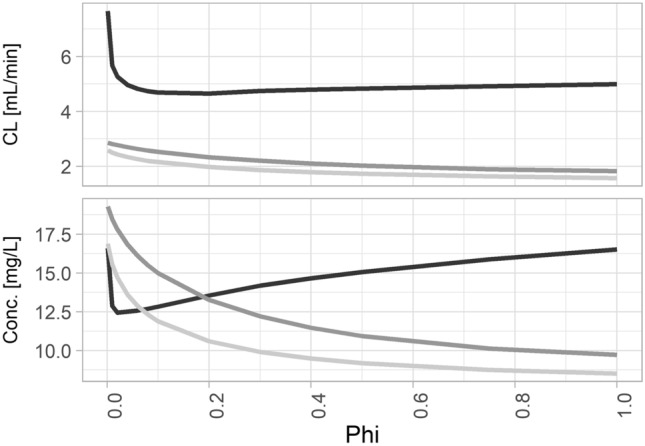
Effect of the prior distribution as defined by the population pharmacokinetic model on RMSE concerning clearance (CL) and iohexol concentration predictions 1440 min post injection (Conc.) The three-compartment model with a simplified random effects structure and with (light gray) or without (dark gray) covariates performed best with an undiscounted prior ($\phi =1$). Both models performed better than the three-compartment model with random effects for all pharmacokinetic parameters (black).

All two-compartment models and the three-compartment model with random effects for all PK parameters revealed a clear relationship between eGFR based on the BIS2 equation^10^ and the prediction error concerning concentrations after 1440 min (Fig. 2). With decreasing eGFR values, predictions and observations differed increasingly. In contrast, the three-compartment model with limited random effects showed only a slight increase in prediction error at low eGFR values and the corresponding model with covariates indicated no relevant relationship between eGFR and the adequacy of predictions. Full support by the population model ($\phi =1$) provided the lowest RMSE in the case of the simplified three-compartment models (with and without covariates), while a reduced model impact (e.g., $\phi =0.01\,to\,0.1$) provided the lowest RMSE when using the three-compartment model with random effects for all PK parameters (Fig. 3). The simplified three-compartment model provided the lowest overall RMSE, i.e., a relevantly lower RMSE than the best full three-compartment model.

### Part III—identification of optimal sampling times

**Figure 4:**
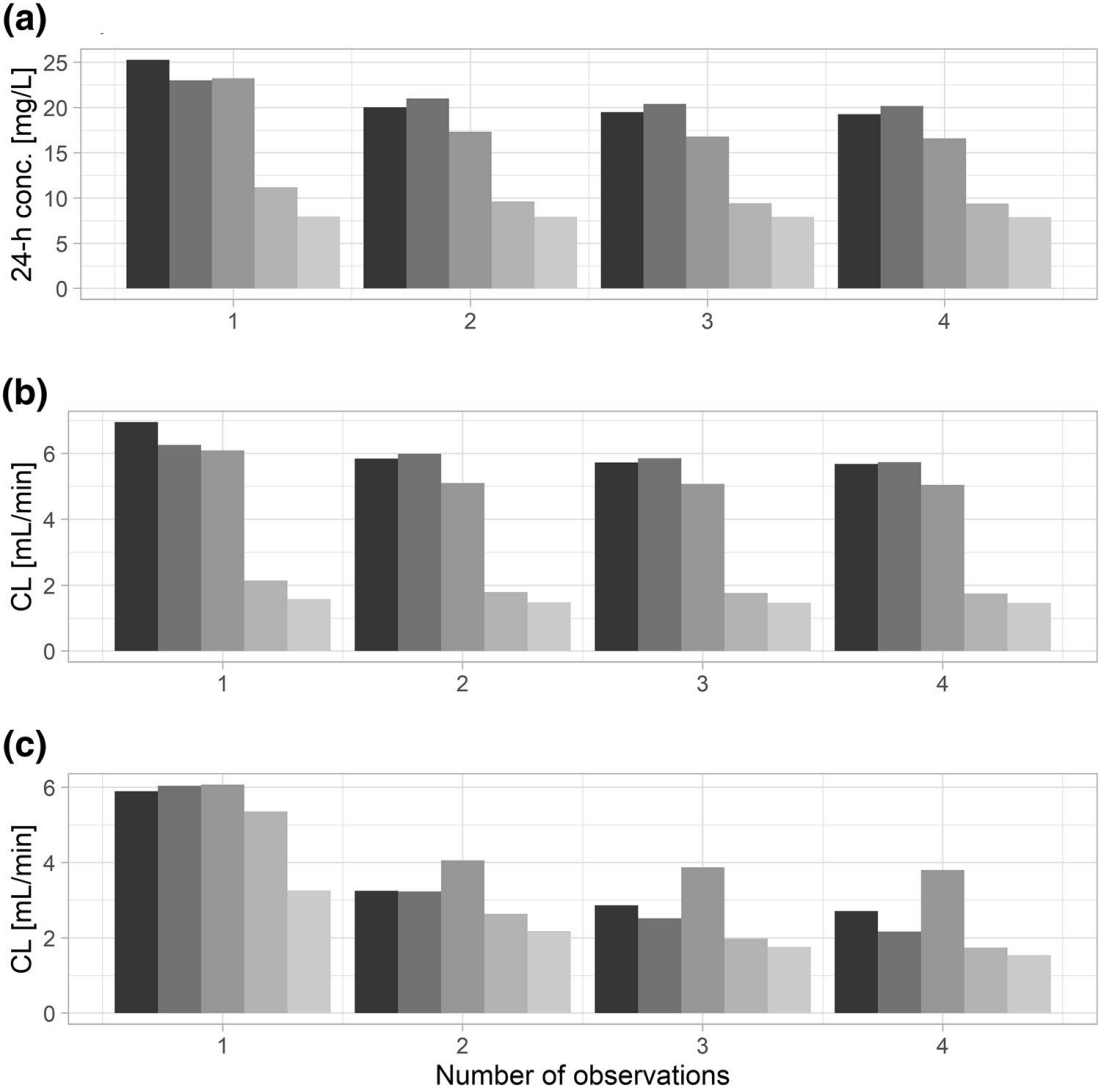
RMSE concerning predicted versus observed iohexol concentrations 1440 min post injection (**a**) and concerning clearance (CL) based on observed data (**b**) and stochastic simulations (**c**). Different shades of gray represent the full two-compartment, reduced two-compartment, full three-compartment, reduced three-compartment, and the reduced three-compartment model with covariates (from left to right/black to light gray).

Optimal iohexol sampling times for different models and with one to four samples per patient are shown in Table 2 (based on observed data) and Table 3 (based on stochastic simulations). Based on the observed data, the sample 300 min post injection provided the most pronounced improvement in terms of RMSE (Table 2). This was further improved when including an earlier sample obtained 2 h post injection. Increasing the number of samples from a single to two samples provided a clear decrease in RMSE for most models (Fig. 4a, b). In contrast, the availability of three or more samples provided only a small further decrease in RMSE. In the presence of covariates, differences in RMSE with one to four samples were marginal.

**Table 2:** Combinations of sampling times with N = 1 to 4 samples providing the lowest root mean squared error concerning clearance based on observed data.

Model	Optimal sampling times [minutes post injection]			
	N = 1	N = 2	N = 3	N = 4
2 cmt, full	300	120, 300	30, 120, 300	30, 120, 240, 300
2 cmt, limited	300	120 (30), 300	30, 240 (120), 300	30, 60 (120), 240, 300
3 cmt, full	300	120, 300	120 (30), 180 (120), 300	120, 180, 240, 300
3 cmt, limited	300	120, 300	30 (60), 120, 300	30 (120), 60 (180), 120 (240), 300
3 cmt, limited, cov	120	120, 240 (300)	60 (90), 120, 150 (300)	60 (90), 90 (120), 120 (150), 150 (300)

Brackets indicate optimal sampling times concerning predictions 1440 min post injection if they deviated from clearance-based results.

**Table 3:** Combinations of sampling times with N = 1 to 4 samples providing the lowest root mean squared error concerning clearance based on stochastic simulations.

Model	Optimal sampling times (minutes post injection)			
	N = 1	N = 2	N = 3	N = 4
2 cmt, full	240	60, 300	30, 120, 300	30, 120, 240, 300
2 cmt, limited	180	60, 300	30, 240, 300	30, 180, 240, 300
3 cmt, full	240	60, 300	30, 90, 300	30, 90, 240
3 cmt, limited	180	30, 300	30, 240, 300	30, 180, 240, 300
3 cmt, limited, cov	180	90, 300	30, 240, 300	30, 180, 240, 300

Inclusion of more than one sample had only a marginal impact on CCC and TDI_90_ in case of the limited three-compartment model with and without covariates (Supp. Fig. 4). The CCC improved from 0.97 to 0.98 and the TDI_90_ improved from 16 to 15% when including two samples instead of one with the limited three-compartment model without covariates. The inclusion of further samples did not improve the CCC or TDI_90_. For the three-compartment model with covariates, the CCC was 0.99 and the TDI_90_ was 13% irrespective of the number of samples. In the sub-group of patients with an estimated iohexol clearance ≥ 20 mL/min, the CCC increased from 0.95 with one to 0.97 with two samples, while the TDI reduced from 11% with one to 8.6% with two samples using the limited three-compartment model without covariates. Additional samples provided no further improvement. Based on the model with covariates, the CCC was 0.97 with one to four and the TDI_90_ was 7.3% with one to three samples, with a marginal additional reduction to 7.0% when including four samples. In contrast, the inclusion of at least two samples compared to a single sample had a pronounced impact on CCC and TDI_90_ in case of the full two- and three-compartment models. For example, the CCC improved from 0.79 to 0.86 for the full two- and from 0.81 to 0.87 for the full three-compartment model when including two samples instead of one. Similarly, the TDI_90_ improved from 61 to 50% and from 55 to 40%, respectively.

**Figure 5:**
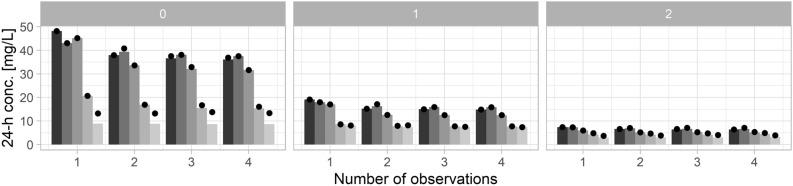
RMSE concerning predicted versus observed iohexol concentrations 1440 min post injection stratified by eGFR quartiles. Different shades of gray represent the full two-compartment, reduced two-compartment, full three-compartment, reduced three-compartment, and the reduced three-compartment model with covariates (from left to right/black to light gray). Patients with low (0), intermediate (1), high (2) eGFR as defined by quartiles (< 25%, corresponding to < 23 mL/min; 25–75%, 23 to 34 mL/min; > 75%, > 34 mL/min). Bars indicate RMSE for optimal sampling times stratified by eGFR while dots represent RMSE in the respective eGFR group when a single sampling protocol is used for all patients.

Optimal sampling times based on stochastic simulations were similar, but favored earlier samples obtained 300 min post injection (Table 3). The inclusion of a second sample provided a relevant decrease in RMSE, while further samples had marginal effects (Fig. 4c). Finally, the eGFR was calculated with the creatinine- and cystatin C-based BIS2 equation to optimize sampling times in different eGFR groups. Optimizing iohexol sampling times separately in the groups of patients with low, intermediate and high eGFR, as defined by the quartiles of the patient collective (low: < 23 mL/min, intermediate: 23 to 34 mL/min, high: > 34 mL/min), provided only a marginally improved RMSE in terms of predictions 1440 min post injection (Fig. 5) and clearance (not shown). Differences in CCC and TDI_90_ between models were marginal in the evaluation based on simulated data (Supp. Fig. 5). While the CCC was at least 0.98 for one to four samples in all cases, the TDI_90_ improved more clearly when including at least two samples compared to one sample (Supp. Fig. 5).

Overall, the limited three-compartment model with covariates provided the best performance in terms of RMSE, CCC and TDI_90_ with only limited improvements when increasing the number of samples beyond two based on observed and simulated data. To achieve the CCC and TDI_90_ goals, two samples were sufficient when using the three-compartment model with covariates (creatinine and cystatin C concentrations, total body weight, age, gender) in patients with an estimated iohexol clearance ≥ 20 mL/min.

## Discussion

A three-compartment model with a simplified random effects structure and with covariates provided the best iohexol clearance estimates in our study sample of 104 individuals aged 70 + and with a mean eGFR of 29.4 mL/min/1.73m^2^. Two iohexol measurements, obtained shortly after injection and 300 min post injection, appear to be sufficient to precisely estimate iohexol clearance using the presented model in patients with impaired kidney function. In contrast to a previous evaluation based on a classical approach^11^, iohexol concentrations measured 1440 min post injection were of minor importance when using the simplified three-compartment model with covariates, which is of great advantage in clinical practice.

Discussions on optimal iohexol protocols, including the choice of a pharmacokinetic model and the number and timing of blood samples, are ongoing. Criteria relevant to the selection of an optimal protocol include precision and accuracy, operating expenses, feasibility and comprehensibility for its users. The requirement for a larger number of late samples (one day after administration of iohexol) would essentially preclude the use of such protocols in most clinical settings. The use of complex models though requires permanent provision of mathematical expertise.

A general issue associated with empirical Bayesian estimation, which forms the basis of estimating iohexol clearance using a population PK model, is shrinkage^23^. In cases where the PK model contributes stronger information than individual iohexol data, for example, due to a limited number of informative blood samples, PK estimates might shrink towards the typical values (geometric means) in the population. Consequently, estimates might be relevantly biased. However, a strong advantage of Bayesian estimation is its capability to reduce the variance of parameter estimates. This plays a pivotal role for three-compartment models since the combination of three exponential terms provides a highly flexible PK model with hardly identifiable parameters if limited data are available from only a single subject. Thus, either extensive blood sampling or the support by a population PK model is needed to warrant reliable PK parameter estimates in the case of three-compartment models. For example, Frennby et al. estimated individual parameters of a three-compartment model in pigs based on 16 plasma samples obtained over a period of 4.5 h^9^, which would not be a reasonable option in clinical practice. The present investigation revealed that the Bayesian procedure provides reasonable iohexol clearance estimates if a limited number of plasma samples was obtained. This improved description of iohexol pharmacokinetics via a three-compartment model outweighs the potential drawbacks associated with EBE estimation.

The feasibility of limited sampling strategies with one to three plasma samples has been evaluated in numerous studies^24,25^. Overall, two or even a single plasma sample was found to be sufficient for obtaining precise clearance estimates^7^. Previous studies suggested blood samples at 2 and 4 h post injection, with delayed sampling after 5 to 8 h post injection in CKD patients^25^. In contrast, we found that the combination of an early sample (e.g., 30 min post injection) with a sample obtained after 300 min appears to be sufficient to precisely estimate iohexol clearance. The shortened post injection period is an important and clinically relevant advantage for implementing iohexol measurement in routine diagnostics.

A sampling scheme based on two samples missed the strict TDI_90_ criterion of ≤ 10% in the sub-group of patients with an estimated iohexol clearance < 20 mL/min. In this sub-group, a late sample obtained > 300 min post injection would be needed to reduce the relative difference between estimated and reference clearance values to at most 10% in 90% of the patients. However, it appears unlikely that a TDI_90_ of 10–20% is of high clinical relevance in patients with a significantly reduced kidney function. For example, the TDI_90_ observed in patients with a clearance < 20 mL/min and based on the limited three-compartment model with covariates amounted to 15%. This implies that the clearance estimates for 90% of all evaluated patients with a true clearance of 20 mL/min would range from 17 to 23 mL/min, with very limited impact on clinical decision making. Principally, the need for an additional late sample could be assessed on the fly if the bioanalytical quantification of iohexol and the Bayesian calculations were carried out rapidly after obtaining the first blood samples. This method would allow to use an intermediate iohexol clearance estimate to assess whether additional samples should be collected, but it appears questionable whether the limited expected improvement in clearance estimates justifies the increased complexity of this approach.

Slight differences in optimal sampling schemes compared to previous evaluations might relate to the three-compartment approach. More specifically, if a two-compartment model tends to provide over-estimated clearance values due to a distributional component that especially affects early concentrations^6^, late observations might be needed to obtain appropriate estimates. This is highlighted by the finding that samples obtained during the late elimination phase were not needed in terms of RMSE when using a simplified three-compartment model, while late samples improved clearance estimates when using a two-compartment approach. Importantly, the employed population pharmacokinetic models include an estimate of the random error associated with observed iohexol plasma concentrations. The magnitude of this random error might change depending on the setting, including potential differences between hospitals and laboratories. If the magnitude of random error increases, more samples than identified in our evaluation might be needed. A similar argumentation applies to scenarios in which the sample collection and/or the quantification of iohexol concentrations is expected to be exceptionally error prone. Then, the collection of more than two samples might be preferrable to ensure that sufficient data is available to estimate iohexol clearance.

Finally, we could not evaluate whether a sample obtained > 300 and < 1440 min post injection would be beneficial since no observations from this time window were available. This might be of particular interest for patients with a very low GFR. For example, Gaspari et al. identified an optimal sampling time of 10 h post injection for subjects with a low GFR, while additionally emphasizing that a one-sample approach might lead to unacceptable errors compared to a multiple sample approach^26^. Due to the remaining ambiguities and the heterogeneity of results of different studies with respect to one-sample schedules, the inclusion of at least two observations might be preferable compared to a one-sample approach.

The inclusion of covariates further improved clearance estimates. Patients with reduced eGFR may benefit relevantly from the inclusion of covariates (cystatin C, creatinine, weight, age, and sex) particularly when only a single iohexol sample is available. However, the positive effects of creatinine and cystatin C observations on iohexol clearance estimates might be limited in clinical scenarios with a strong GFR dynamic, such as acute kidney injury, due to a discrepancy between creatinine and cystatin C concentrations with actual GFR. To allow for the implementation of models with and without covariates, the corresponding model parameter estimates are presented in Supp. Table 1.

Since the population PK model plays a major role in the estimation of individual clearance, state-of-the art approaches should be used for a proper model evaluation. This includes the evaluation of visual predictive checks, which allow assessing whether the entire model structure, including distributional assumptions on PK parameters, matches the observed data sufficiently. While this applies to the models presented in our evaluation (Supp. Fig. 2), other recently published models do not fulfil this criterion successfully. For example, Åsberg et al.^4^ recently reported that a two-compartment model performed best, which we think is not confirmed by the VPCs presented by the authors. A misspecified model might have detrimental effects on clearance estimates, particularly when working with a small number of individual samples. For the use of our model, it should be noted that patients in the Berlin Initiative Study were at least 70 years old. Thus, it remains to be shown whether our findings can be transferred to estimate iohexol clearance in young(er) patients. Furthermore, the determination of optimal sampling times was confined to the available sampling times plus observations 6 and 8 h post injection in the stochastic simulations. It cannot be precluded that other sampling times would be superior and classical optimal design approaches, for example based on D_s_-optimality^27^, might be useful to further optimize sampling protocols. However, the evaluated sampling times cover most of the previously evaluated designs^25^. While concentrations observed 1440 min post injection were used as a main tool to validate models in this evaluation, other approaches of model validation might also be useful. This could comprise a comparison of model-based iohexol clearance (and, therefore, mGFR) estimates and iohexol clearance estimates obtained based on either extensive plasma sampling (AUC-based estimation) or plasma sampling combined with urine collection (renal clearance-based estimation). Given the respective data, iohexol clearance were determined without compartmental assumptions, providing model-independent reference values to compare different clearance estimation approaches.

In conclusion, we suggest a formula based on a simplified three-compartment model including covariates to estimate iohexol clearance in elderly patients with reduced GFR. Two iohexol plasma samples, taken shortly after administration and 300 min post injection, are sufficient to estimate iohexol clearance adequately based on this model. Future development of model-based iohexol approaches should include younger patients and different ethnicities, expanding this model-based approach to patients in all clinical settings. Noteworthy limitations of this evaluation might be the lack of data from young(er) patients and patients with normal kidney function, the lack of iohexol data obtained between 300 and 1440 min post injection, and the observed slightly lower performance in patients with a iohexol clearance < 20 mL/min.

## Supplementary Information

Supplementary Information 1.

Supplementary Information 2.

## Data Availability

The datasets analyzed during the current study are available from the corresponding author on reasonable request.
