# Supplementary material for: Advancement of pharmacokinetic models of iohexol in patients aged 70 years or older with impaired kidney function
Source: Sci Rep. 2021 Nov 22;11:22656. doi: 10.1038/s41598-021-01892-1 (PMC8608910; doi:10.1038/s41598-021-01892-1)
Supplement: Supplementary file 1 — Supplementary Information 1. [file 41598_2021_1892_MOESM1_ESM.docx]

**Advancement of pharmacokinetic models of iohexol in patients with impaired kidney function**

Max Taubert^1^, Elke Schaeffner^2^, Peter Martus^3^, Markus van der Giet^4^, Uwe Fuhr^1^, Amina Lösment^5^ & Natalie Ebert^2^

1 University of Cologne, Faculty of Medicine and University Hospital Cologne, Department I of Pharmacology, Cologne, Germany

2 Institute of Public Health, Charité – Universitätsmedizin Berlin, Berlin, Germany

3 Institute for Clinical Epidemiology and Applied Biostatistics, University Hospital Tübingen, Tübingen, Germany

4 Department of Nephrology, Charité – Universitätsmedizin Berlin, Berlin, Germany

5 Department of Nephrology, Vivantes Klinikum im Friedrichshain, Berlin, Germany

**Supplementary Figures**

**Flow chart with the selection process of the study population**

| BIS population **n = 2069** |  | Inclusion criteria for iohexol plasma clearance measurement:  - age 70 and above  - thyroid-stimulating   hormone level >0.3 mIU/L  - no known iodine allergy | | | | |  | Patients with considerably impaired kidney function transferred from specialist care (serum creatinine level of ≥1.5 mg/dL)  **n = 87** |
| --- | --- | --- | --- | --- | --- | --- | --- | --- |
| Subset with iohexol plasma clearance measurement  **n = 570**  (Schaeffner et al. 2012) |  |  |  |  |  |  |  |  |
|  |  |  | | | | |  |  |
| Selection of patients with plausible iohexol data for population PK modeling  **n = 563** (Taubert et al. 2018) |  | Excluded due to implausible iohexol data  **n = 7** | | | | |  |  |
|  |  |  | | | | |  |  |
|  |  | Subset:  considerably impaired kidney function (serum creatinine level of ≥1.5 mg/dL)  **and**  valid iohexol plasma clearance measurement over 1440 min. post injection  **n = 17** | | | | |  | Subset:  valid iohexol plasma clearance measurement over 1440 min. post injection  **n = 87** |
|  |  |  | | | | |  | 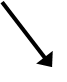 |
| Model development group with iohexol data up to 300 minutes post injection  **n = 546** | | |  |  |  | Evaluation group with iohexol data up to 1440 minutes post injection  **n = 104** (Ebert et al. 2015) | | |

**Supp. Figure 1.** Flow chart with the selection process of the study population.

**Visual predictive checks for the final population pharmacokinetic models**


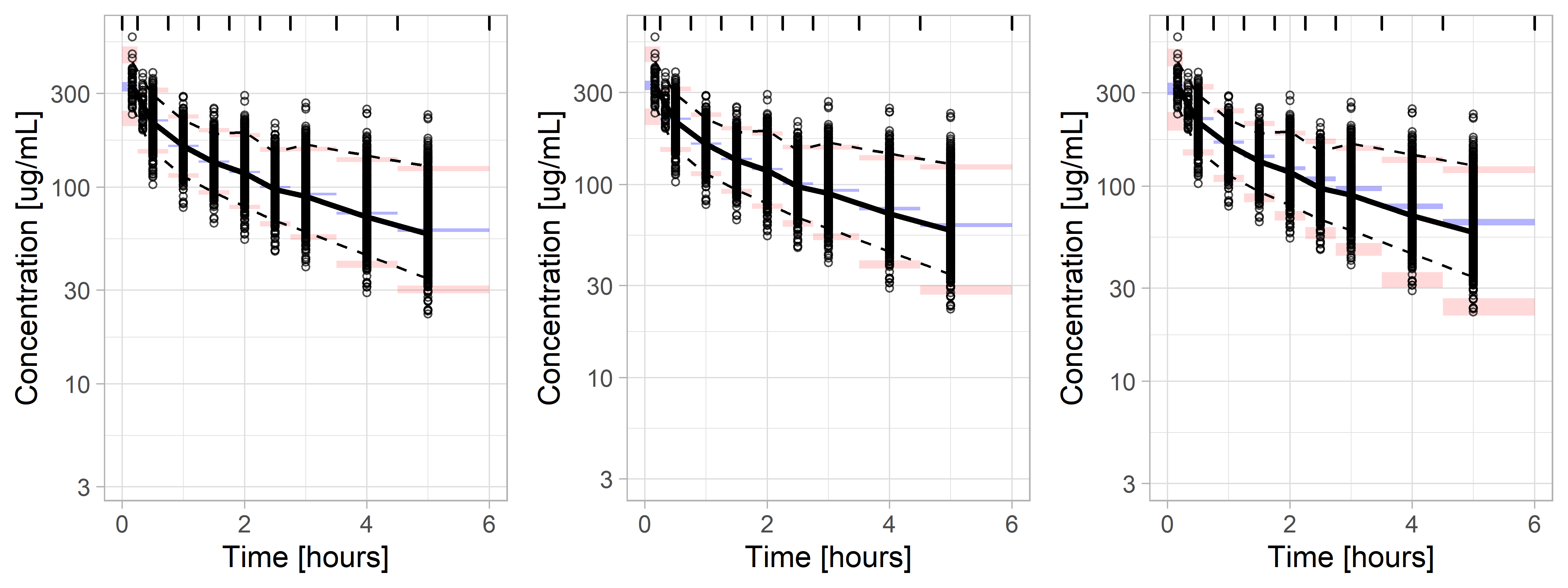


**Supp. Figure 2**. Visual predictive checks (VPC) corresponding to the final population pharmacokinetic three-compartment models with covariates, without covariates and with all covariates but cystatin C. Median (solid line) and 90% interval (dashed lines) of observed concentrations as well as 90% confidence intervals of the simulated median and prediction intervals based on the model (boxes).

**Total deviation index for the evaluated models**


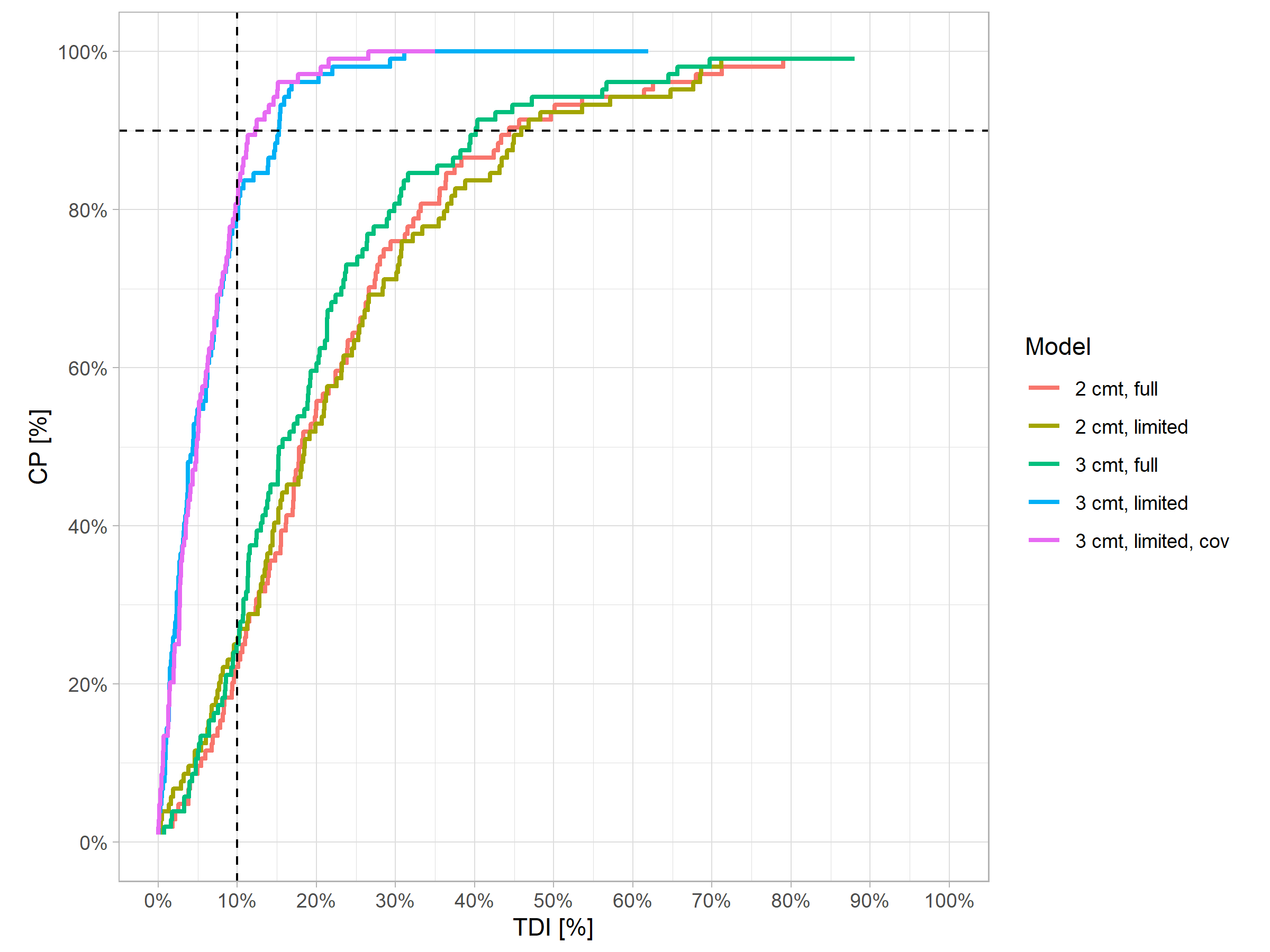


**Supp. Fig. 3.** Empirical distribution showing the Total Deviation Index (TDI) for a range of Coverage Probabilities (CP). The TDI shows the relative deviation between clearance estimates obtained with versus without observations 1440 minutes post injection. The CP is the corresponding percentage of patients not exceeding the TDI. The dashed lines indicate TDI_90_ (horizontal line) and CP_10_ (vertical line).

**Additional measures describing the concordance of GFR estimates given a varying number of included iohexol observations based on data observed in patients**


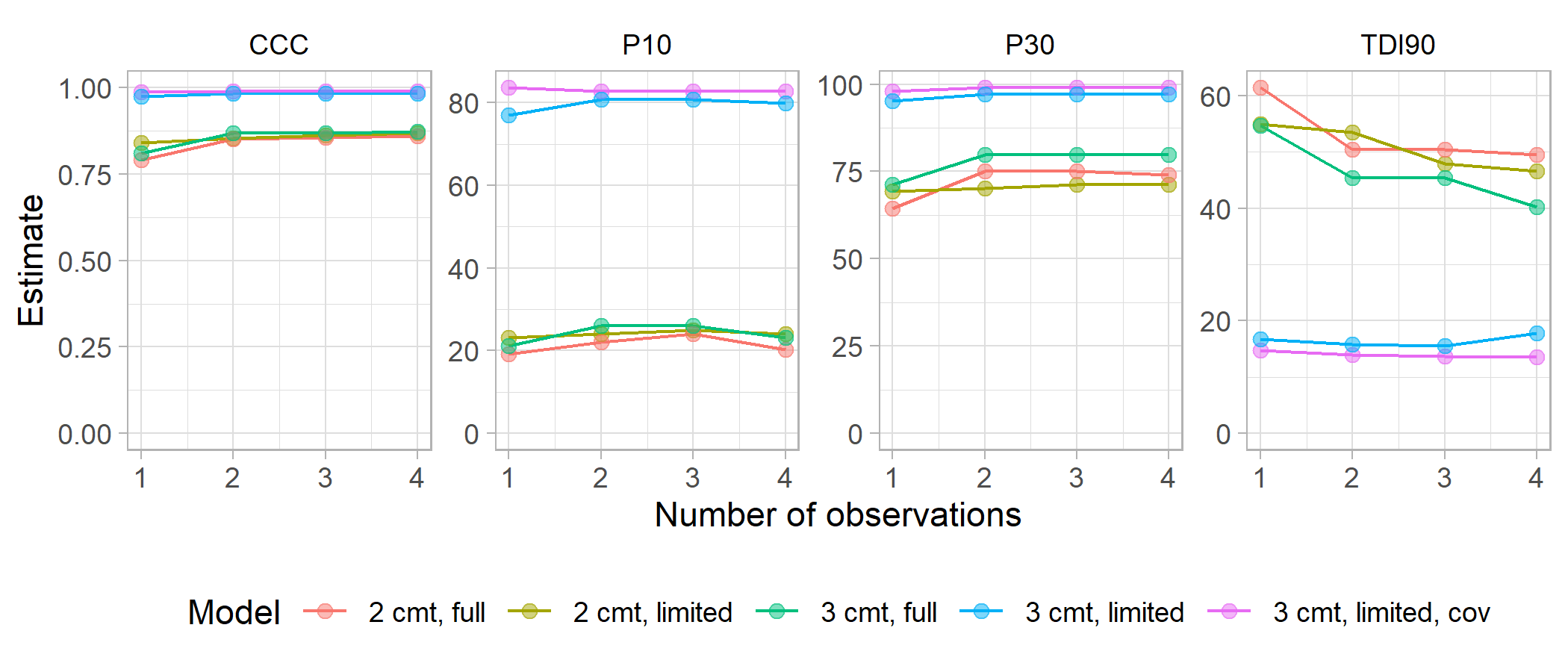


**Supp. Fig. 4.** Additional measures describing the concordance between GFR estimates based on 1 to 4 samples up to 300 minutes post injection versus estimates obtained with all available samples including 1440 minutes post injection based on iohexol data observed in patients. Concordance correlation coefficient (CCC); percentage of relative deviations within 10% (P10) and within 30% (P30) of the reference values; Total Deviation Index for a coverage probability of 90% (TDI_90_).

**Additional measures describing the concordance of GFR estimates given a varying number of included iohexol observations based on simulated data**


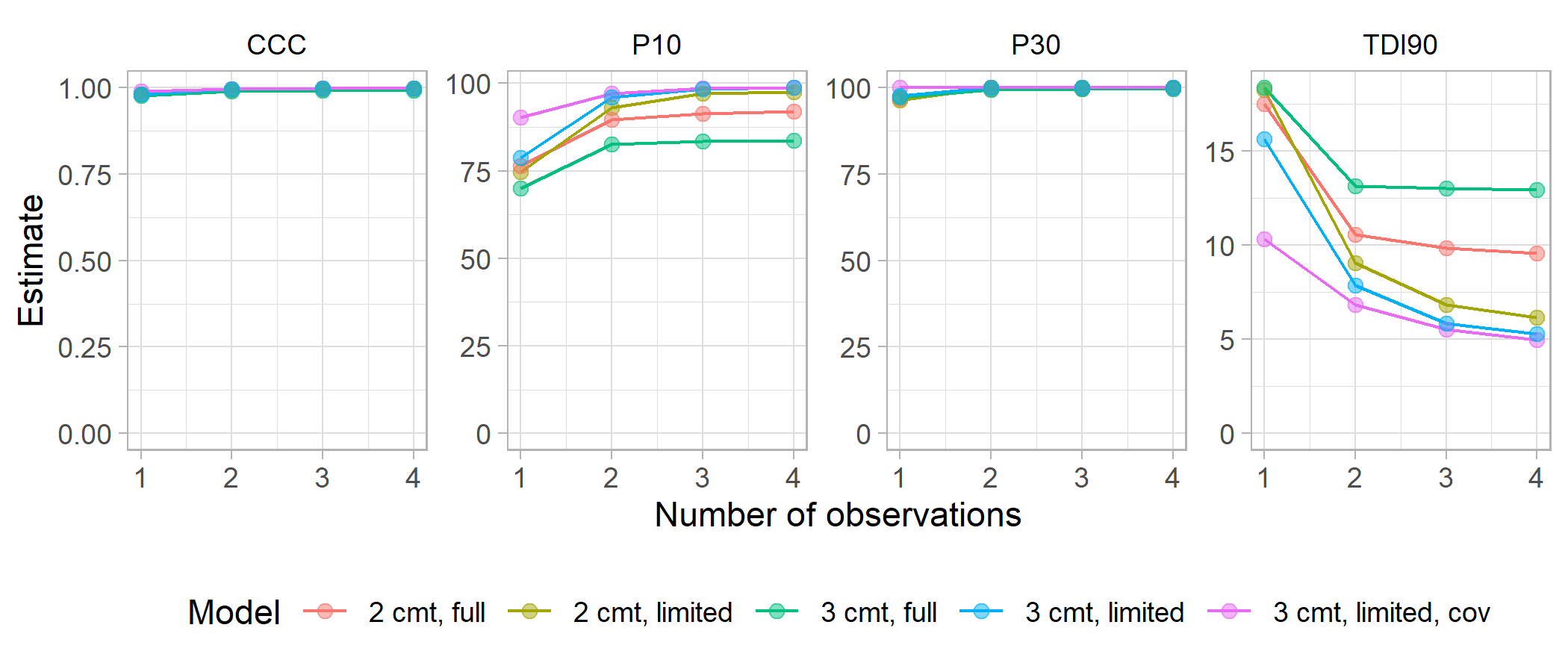


**Supp. Fig. 5.** Additional measures describing the concordance between GFR estimates based on 1 to 4 samples up to 300 minutes post injection versus estimates obtained with all available samples including 1440 minutes post injection based on iohexol data simulated from the respective model. Concordance correlation coefficient (CCC); percentage of relative deviations within 10% (P10) and within 30% (P30) of the reference values; Total Deviation Index for a coverage probability of 90% (TDI_90_).
