# Supplementary material for: Advancement of pharmacokinetic models of iohexol in patients aged 70 years or older with impaired kidney function
Source: Sci Rep. 2021 Nov 22;11:22656. doi: 10.1038/s41598-021-01892-1 (PMC8608910; doi:10.1038/s41598-021-01892-1)
Supplement: Supplementary file 2 — Supplementary Information 2. [file 41598_2021_1892_MOESM2_ESM.docx]

**Advancement of pharmacokinetic models of iohexol in patients with impaired kidney function**

Max Taubert^1^, Elke Schaeffner^2^, Peter Martus^3^, Markus van der Giet^4^, Uwe Fuhr^1^, Amina Lösment^5^ & Natalie Ebert^2^

1 University of Cologne, Faculty of Medicine and University Hospital Cologne, Department I of Pharmacology, Cologne, Germany

2 Institute of Public Health, Charité – Universitätsmedizin Berlin, Berlin, Germany

3 Institute for Clinical Epidemiology and Applied Biostatistics, University Hospital Tübingen, Tübingen, Germany

4 Department of Nephrology, Charité – Universitätsmedizin Berlin, Berlin, Germany

5 Department of Nephrology, Vivantes Klinikum im Friedrichshain, Berlin, Germany

**Supplementary Tables**

|  | **With covariates** | | **Without cystatin C** | | | **Without covariates** | | |
| --- | --- | --- | --- | --- | --- | --- | --- | --- |
| **Parameter** | **Estimate** | **RSE [%]** | | **Estimate** | **RSE [%]** | | **Estimate** | **RSE [%]** |
| CL [L/h] | 3.81 | 1 | | 3.90 | 1 | | 2.97 | 2 |
| V1 [L] | 7.75 | 4 | | 7.72 | 4 | | 7.67 | 4 |
| Q1 [L/h] | 2.02 | 5 | | 2.03 | 5 | | 2.04 | 4 |
| V2 [L] | 4.84 | 3 | | 4.86 | 3 | | 5.03 | 3 |
| Q2 [L/h] | 6.42 | 12 | | 6.52 | 12 | | 6.70 | 11 |
| V3 [L] | 2.62 | 8 | | 2.65 | 8 | | 2.72 | 8 |
| Creatinine [mg/dL] | -0.455 | 9 | | -1.09 | 2 | | --- | --- |
| Cystatin C [mg/L] | -0.748 | 5 | | --- | --- | | --- | --- |
| Age [years] | -0.664 | 12 | | -1.06 | 10 | | --- | --- |
| Sex (female) | -0.120 | 12 | | -0.197 | 8 | | --- | --- |
| Weight (CL) | 0.827 | 5 | | 0.789 | 7 | | --- | --- |
| Weight (V1) | 0.890 | 5 | | 0.892 | 5 | | --- | --- |
| $\Omega_{1,1}$ (CL) | 0.0219 | 7 | | 0.0356 | 9 | | 0.225 | 7 |
| $\Omega_{2,2}$ (V1) | 0.0356 | 8 | | 0.0358 | 8 | | 0.0622 | 7 |
| $\Omega_{1,2}$ (cov.) | 0.0140 | 11 | | 0.0144 | 13 | | 0.0517 | 11 |
| $\Sigma_{1,1}$ (prop.) | 1.00e-3 | 9 | | 9.70e-4 | 9 | | 9.60e-4 | 9 |
| $\Sigma_{2,2}$ (add.) | 3.05 | 23 | | 3.16 | 22 | | 3.26 | 22 |

**Supp. Table 1**. Parameter point estimates and relative standard errors (RSE) obtained from NONMEM. Model with all covariates (left), without cystatin C (middle) and without covariates (right). Variance ($\Omega_{1,1}$, $\Omega_{2,2}$) and covariance ($\Omega_{1,2}$) of random effects and the variance of the proportional ($\Sigma_{1,1}$) and additive ($\Sigma_{2,2}$) residual error terms corresponding to $y_{ij}=IPRED_{ij} \times\left( 1+\epsilon_{1,1} \right)+\epsilon_{2,2}$, where $y_{ij}$ is the j^th^ observation in the i^th^ subject and $IPRED_{ij}$ is the predicted concentration of iohexol[µg/ml]. Models fitted to data from previous and additional patients, i.e., including 667 patients in total.

| **Model** | **CCC** | **TDI_90_** | **P10** | **P30** |
| --- | --- | --- | --- | --- |
| 3 cmt, limited, covariates | 0.99 | 12% | 80% | 99% |
| 2 cmt, limited | 0.87 | 47% | 24% | 70% |
| 3 cmt, limited | 0.98 | 15% | 78% | 98% |
| 2 cmt, full | 0.86 | 45% | 21% | 75% |
| 3 cmt, full | 0.87 | 40% | 24% | 80% |

**Supp. Table 2.** Additional measures describing the concordance between GFR estimates with versus without observations 1440 minutes post injection. Concordance correlation coefficient (CCC); Total Deviation Index for a coverage probability of 90% (TDI_90_); percentage of relative deviations within 10% (P10) and within 30% (P30) of the reference values.
